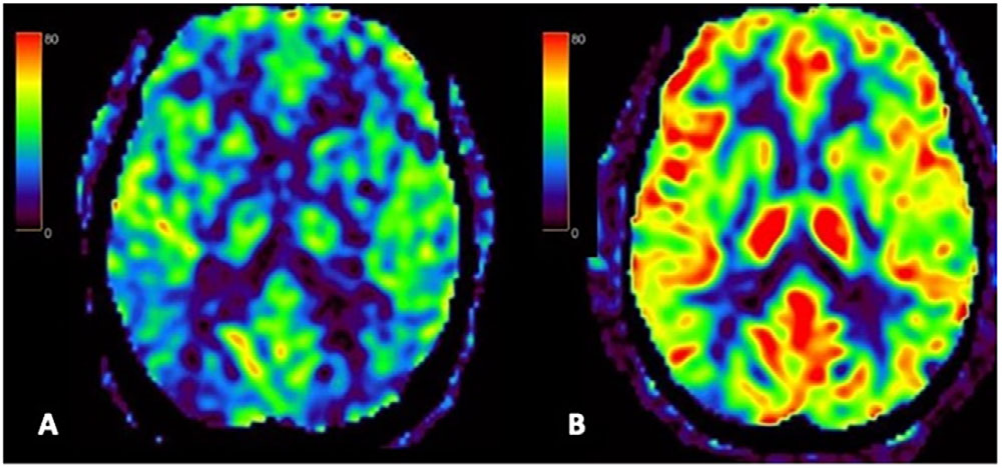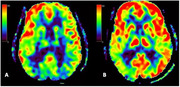# Increased cerebral blood flow after lecanemab infusions: MR‐ASL utility in monitoring Alzheimer disease treatment response

**DOI:** 10.1002/alz.095803

**Published:** 2025-01-09

**Authors:** Anna S Nordvig, Andres Ricaurte Fajardo, Gloria Chiang

**Affiliations:** ^1^ Weill Cornell Medicine, New York City, NY USA

## Abstract

**Background:**

Since lecanemab, an anti‐amyloid monoclonal antibody targeting Aβ protofibrils, only modestly impacts cognition (27% slower cognitive decline over 18 months), tracking the effects on the brain in the early months is a clinical challenge. Serial noncontrast MRI scans are required by the FDA, to assess for the occurrence of amyloid‐related imaging abnormalities (ARIA). Arterial spin‐labeling MR (ASL‐MR) is a 4‐minute non‐contrast easily‐added sequence. Patterns of abnormal cerebral blood flow (CBF) on ASL‐MR can correlate with neurodegenerative markers including FDG‐PET.

**Method:**

Here we show CBF maps of two patients at baseline, and after receiving 4 infusions of lecanemab. Our figure is representative of six other patients in whom we have noted similar changes on therapy, and who will also be presented.

**Result:**

Figure 1 shows CBF maps of a man in his early seventies with mild cognitive impairment, due to AD. He had a family history of AD, and was diagnosed with a positive 18F‐florbetaben PET and prescribed donepezil, with mild symptom improvement. His CSF markers were borderline (Abeta42 575 pg/mL, t‐tau 289 pg/mL, p‐tau 51 pg/mL, ATI 0.99.). Figure 2 shows CBF maps of a woman in her fifties, with a history of Crohn’s disease, not currently requiring immunosuppression. She was diagnosed with posterior cortical atrophy due to AD, with a positive 18F‐florbetaben PET and CSF compatible with AD (abeta42 366 pg/mL, t‐tau 486 pg/mL, p‐tau 72 pg/mL, ATI 0.45). She remained employed until a mild COVID infection, at which point her preexisting cognitive symptoms worsened (presumed longCOVID‐related worsening). Both patients carried the ApoE e3/e3 genotype. In both cases, baseline low temporoparietal CBF increased at the scheduled MRI post‐4^th^ infusion.

**Conclusion:**

These cases show that CBF increases on lecanemab from baseline to after the 4th infusion. Preclinical investigations have demonstrated that it can protect neurons from Aβ‐induced apoptosis, leading to neuronal viability and improved brain perfusion. CBF increases may also reflect increased synaptic activity after amyloid clearance and/or vascular changes, such as clearance of fibrinogen/amyloid clotting complexes. CBF has potential as a noninvasive and time‐efficient marker of medication effect and perhaps efficacy over time.